# Development of a low-cost upper-body rehabilitation robot for home use

**DOI:** 10.1177/20556683241288226

**Published:** 2024-09-27

**Authors:** David Breton, Thierry Laliberté, Alexandre Campeau-Lecours

**Affiliations:** 1 560498 Centre Interdisciplinaire de Recherche en Réadaptation et Intégration Sociale (Cirris), Quebec City, QC, Canada; 2 Department of Mechanical Engineering, 4440 Laval University, Quebec City, QC, Canada

**Keywords:** Rehabilitation engineering, rehabilitation robotics, motor learning

## Abstract

The inability to use one’s hands or arms greatly restricts the ability to perform daily activities. After a developmental or acquired injury, the intensity and frequency of rehabilitation exercises are essential. To alleviate the burden on the healthcare system, robotic systems have been developed to support clinicians’ interventions. However, these systems are often bulky and expensive, limiting their use to specific clinical settings and making them impractical for home use. This paper presents the development of an affordable and easy to install 2-DOF five-bar linkage robot designed to be used at home. This work aims to reduce the cost of the robot through actuation optimization, mechanical optimization and 3D printing. The architecture and links length are chosen to optimize the robot’s performance in the required workspace. Using sensor feedback, impedance control algorithms and multiple types of exercise such as virtual walls guidance are implemented. Finally, a user interface was programmed to facilitate the robot’s use.

## Introduction

Activities of daily living (AoDL) can be limited by the inability to grasp, manipulate and move objects.^1^ Diverse neurological pathologies such as stroke, spinal cord injury and cerebral palsy can lead to muscular weakness and spasms, which can affect daily activities. Stroke specifically is the leading cause of disability worldwide with risk of suffering a stroke increasing by 50% over the last 17 years.^2^ Following developmental or acquired injury, fast, repetitive and intensive rehabilitation exercises are often necessary to achieve full recovery.^3,4^ However, an anticipated increase in cases and a global labour shortage attributed to rising average age place significant strain on the healthcare system. In response, robotized systems have been developed with the aim to offer exercises to patients and provide feedback to therapists. While these cannot replace conventional therapy, they are useful tools to complement treatment.

Upper-body rehabilitation robots range in complexity with various architecture and degrees of freedom (DoF). Regardless, they can generally be divided in two categories: end effector robots and exoskeleton robots.^5^

End-effector type robots typically consist of a serial or parallel robot with a graspable handle or fixture at the end. By controlling the position of the effector, the robot leads the movement of the limb attached to the effector. For instance, the MIT Manus is a planar 2-DoF five-bar linkage parallel robot with a handle end effector allowing the user to control the robot.^6^ Force based algorithms allow the robot to guide the user along a trajectory. The robot can only be handled by one arm at a time. However, its architecture is asymmetrical, which results in asymmetrical manipulability and force application across the workspace. Given the body’s inherent symmetry along the sagittal plane, the disparate experience encountered with the left and right hands is undesirable. To circumvent this issue, some robots such as the KinArm^7^ opt to duplicate the end effector, enabling simultaneous utilization of both arms. Others have opted to restrict their design to solely symmetrical architectures such as the serial robot REAplan^8^ which uses slider to offer a perfect manipulability in its workspace.

An exoskeleton robot is characterized by an architecture mirroring human anatomy and acts as an additional skeleton for the user. For instance, ARMin^9^ and L-Exos^10^ are serial robots aiming to encompass and guide the user’s arm. The obvious benefit over end-effector type robots is their ability to control the user’s entire arm. However, this requires adjustments to the links length to suit the user’s physiology, thus adding complexity to the system.^11^ Such serial type robots also require strong motors and rigid structure which is cumbersome and undesirable for human-robot interaction. While other robots such as the Dampace^12^ opted to minimize the need for motor on the arm by using cables and hydraulics, the resulting robot is still relatively complex.

One significant drawback of all these robotic systems is their complexity and high cost, which can range from $50,000 to $250,000. Given their high level of complexity, most of these systems necessitate their own dedicated structures and are commonly sold as bulky trolleys, posing challenges in terms of mobility and storage. Furthermore, their intricate nature demands extensive expertise for effective operation, making them suboptimal for use by therapists and patients alike.^13^ As a result, the demand for home-based solutions emerged, with studies outlining design requirements identified by therapists, including repetitive exercises, guided assistance, ease of use, fixed base, security and intuitive interface.^14^ While robots such as the iCone^15^ aim to fulfill this demand, the utilization of expensive actuators does not make them affordable enough for large-scale use. To achieve widespread adoption, a low-cost alternative is required. Thus, exploring methods such as leveraging consumer-grade electronics and employing manufacturing techniques like 3D printing becomes essential for cost reduction.

This paper presents the development of an affordable and easy to use upper-body rehabilitation robot. The kinematic section details the chosen architecture while the optimisation section presents the results of links length optimization based on this architecture. The design section showcases design solutions for cost reduction and the desired control scheme. The control section presents the control schematic as well as the desired levels of assistance to be programmed into the robot. Finally, the conclusion is presented.

## Kinematics

### Architecture

A common way to minimize cost of robots is to minimize the number of actuators required for the application. Fewer actuators also lead to decreased complexity and size, albeit at the expense of reducing the number of controllable DoF. Exoskeletons, for instance, require many actuators, therefore increasing cost. This, along with other drawbacks already discussed, has directed our search for a low-cost solution towards end-effector type robots.

While most daily activities are three-dimensional, the effectiveness of planar 2-DoF robots has been demonstrated. Indeed, clinical results following MIT-Manus therapy showcased a reduction of impairment in the shoulder and elbow^16^ prompting the adoption of similar exercises by other robotic systems. Furthermore, incorporating a third DoF introduces significantly more complexity compared to the initial two DoFs, rendering it less cost-effective. Thus, a planar 2-DoF architecture is chosen due to its minimal actuator requirement and proven clinical efficacy.

Furthermore, to reduce the inertia at the robot effector, a parallel type architecture is preferred since the actuators are fixed at the base instead of being installed in the link chain. This design decision eliminates the need for moving actuators and sturdier links. This in turns makes the robot easier to move and safer for human interaction.

**Figure 1.**
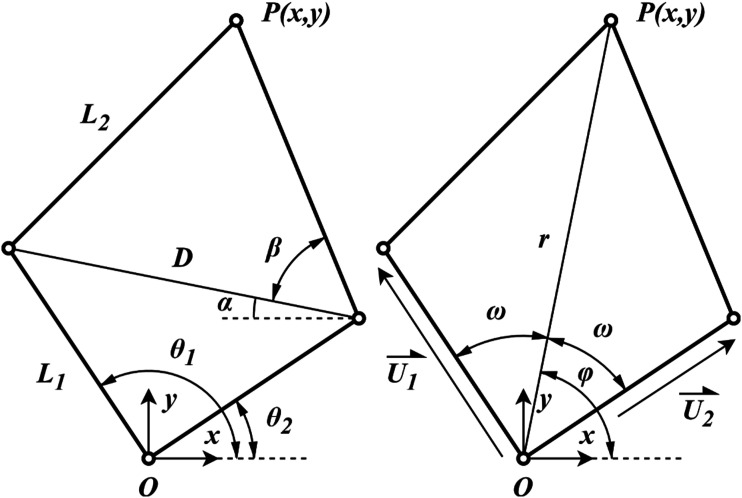
Schematic of the robot’s architecture in desired configuration. The double pivot at *O* is fixed.

The resulting architecture is the 5R linkage 2-DoF parallel robot depicted in Figure 1. In this linkage, the fifth link corresponds to the base fixture. In the development of similar architectures,^17^ it has been determined that the maximum workspace is achieved when the base joints overlap. While this is often impossible due to mechanical constraints, this paper presents a mechanical solution in order to achieve this. The robot’s architecture is symmetrical to allow its use with both arms with similar performance. As a result, both proximal links are of equal length *L*_1_ and both distal links are of equal length *L*_2_.

### Inverse kinematic

The joint variables $\overset{˗}{\theta }={\left[\theta_{1},\theta_{2}\right]}^{T}$ can be expressed as a function of the end-effector position $\overset{˗}{P}={\left[x,y\right]}^{T}$ using geometric relations and Figure 1.

(1) $$\left[\begin{array}{c} \theta_{1}\\ \theta_{2} \end{array}\right]=\left[\begin{array}{c} \varphi +\psi \\ \varphi -\psi \end{array}\right]$$

(2) $$\varphi =\arctan\left(\frac{y}{x}\right)$$

(3) $$\psi =\arccos\left(\frac{L_{1}^{2}+x^{2}+y^{2}-L_{2}^{2}}{2L_{1}\sqrt{x^{2}+y^{2}}}\right)$$

### Forward kinematic

The end-effector position $\overset{˗}{P}={\left[x,y\right]}^{T}$ can be expressed as a function of the joint variables $\overset{˗}{\theta }={\left[\theta_{1},\theta_{2}\right]}^{T}$ . In Figure 1, a line of length *D* is drawn between the intermediate links. Regarding angle *α*, which represents the angle between the drawn line and the *x*-axis, its value as shown is negative.

(4) $$\overset{˗}{P}=\left[\begin{array}{c} L_{1}\cos\left(\theta_{1}\right)+L_{2}\cos\left(\beta -\alpha \right)\\ L_{1}\sin\left(\theta_{1}\right)+L_{2}\sin\left(\beta -\alpha \right) \end{array}\right]$$

(5) $$\alpha =\arcsin\left(\frac{L_{1}\left(\sin\left(\theta_{1}\right)-\sin\left(\theta_{2}\right)\right)}{D}\right)$$

(6) $$\beta =\arccos\left(\frac{D}{2L_{2}}\right)$$

(7) $$D=L_{1}\sqrt{2-2\cos\left(\theta_{1}-\theta_{2}\right)}$$

### Jacobian matrices

Using closed-loop equations, it is possible to obtain both Jacobian matrices as expressed in equation (8).^18^

(8) $$J\dot{\overset{˗}{P}}=K\dot{\overset{˗}{\theta }}$$

(9) $$J=\left[\begin{array}{cc} x-L_{1}\cos\left(\theta_{1}\right) & y-L_{1}\sin\left(\theta_{1}\right)\\ x-L_{1}\cos\left(\theta_{2}\right) & y-L_{1}\sin\left(\theta_{2}\right) \end{array}\right]$$

(10) $$K=\left[\begin{array}{cc} A & 0\\ 0 & B \end{array}\right]$$

(11) $$\begin{array}{c} A=L_{1}\left(y-L_{1}\sin\left(\theta_{1}\right)\right)\cos\left(\theta_{1}\right)\\ -L_{1}\left(x-L_{1}\cos\left(\theta_{1}\right)\right)\sin\left(\theta_{1}\right) \end{array}$$

(12) $$\begin{array}{c} B=L_{1}\left(y-L_{1}\sin\left(\theta_{2}\right)\right)\cos\left(\theta_{2}\right)\\ -L_{1}\left(x-L_{1}\cos\left(\theta_{2}\right)\right)\sin\left(\theta_{2}\right) \end{array}$$

## Geometric analysis

### Linkage length ratio

**Figure 2.**
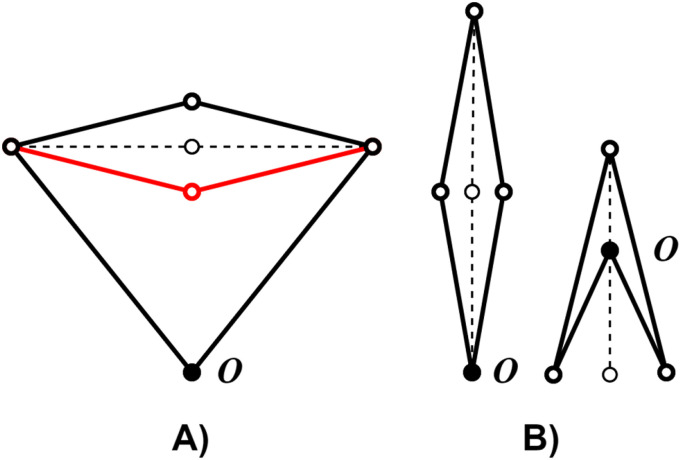
Representation of type I (A) and type II (B) singular configurations. Solid lines represent configurations nearing the singularity, while dotted lines represent the singular configurations. The red configuration represents the second solution branch.

The remaining parameter to consider is the ratio of linkage length *L*_1_/*L*_2_ as pictured in Figure 1. For ratios greater than 1, the mechanism exhibits two kinematic solution branches. These branches are separated by type II singularity, as shown in Figure 2A). To avoid singularity, only one solution branch must be chosen, thereby dividing the workspace and reducing its utility. Otherwise, the workspace is constrained by type I singularity, which represents the maximum reach of the robot or a change in configuration as shown in Figure 2B).

The objective of this analysis is to improve the robot’s dexterity as much as possible. This improvement aims to enhance the robot’s capacity to move consistently in all directions within the workspace. Indeed, the dexterity represents the consistency of the robot’s rigidity along its primary axes. For a dexterity value lower than 1, the robot exhibits greater rigidity along one direction compared to others. Getting this value closer to 1 is therefore crucial for ensuring a feeling of uniformity in robot movements for the user. This value is derived from the conditioning number of the matrix **J′**, representing the ratio between the maximum and minimum singular values of **J′** as expressed in equation (13).

(13) $$D=\frac{1}{cond\left(J^{\prime }\right)}=\frac{\sigma_{\min}}{\sigma_{\max}}$$

where

(14) $$J^{\prime }=J^{-1}K$$

Since both base pivots overlap at *O*, it is possible to analyze this parameter as a function of the distance between base pivot *O* and end effector *P* (i.e. the radius of reachable workspace of the robot). This distance is represented in Figure 1 by *r*. A adimensional analysis is possible by using ratio *r*/*R*_*max*_ where *R*_*max*_ is the maximal radial reach. The conditioning number of the matrix J’ depends on length *r*, leading to changes in angle between both proximal links, but does not depend on the angle *ϕ*. Indeed, in a given configuration, a different angle *ϕ* just pivots the mechanism around *O*.

**Figure 3.**
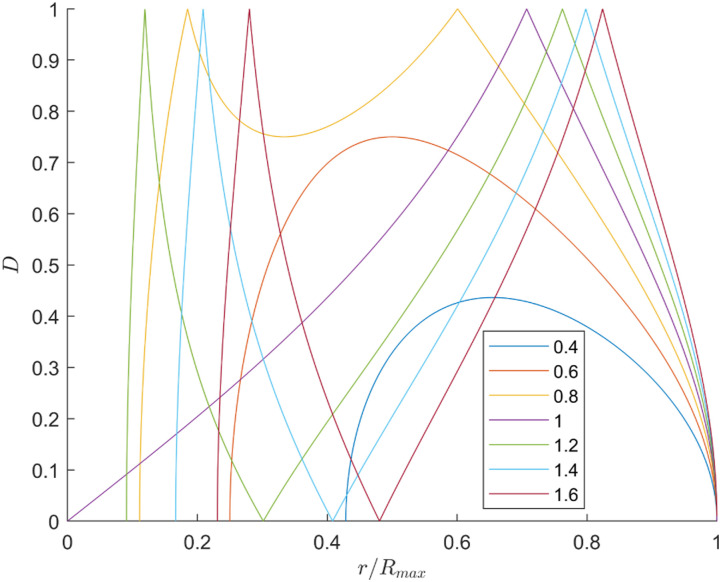
Dexterity of the robot in the radial reachable workspace for multiple ratios *L*_1_/*L*_2_ ranging from 0.4 to 1.6.

Figure 3 illustrates the dexterity in the radial reachable workspace (ratio *r*/*R*_*max*_) for ratios ranging from 0.4 to 1.6. When the ratio is below 1, the maximum dexterity shifts leftward until it reaches 0.707, after which it transitions into a local minimum. This minimum continues to shift leftward until reaching a single peak at a ratio of 1. For ratios above 1, this peak shifts rightward as the ratio increases. Furthermore, the dexterity is separated in two peaks by a point of zero dexterity which corresponds to the type II singularity. Therefore, each peak represents a distinct solution branch.

**Figure 4.**
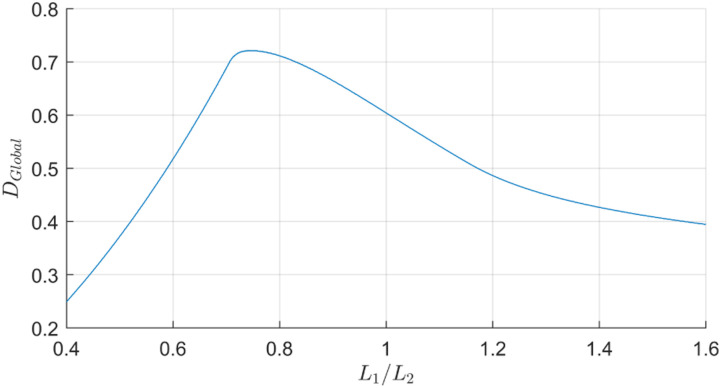
Global dexterity of the robot in the adjusted radial reachable workspace for ratios *L*_1_/*L*_2_ ranging from 0.4 to 1.6.

To better compare the results for multiple ratios, the global radial dexterity is plotted in Figure 4 as a function of the ratio. Global radial dexterity is defined as the mean dexterity value across the radial range. However, to avoid getting close to type I singularities, the angle between proximal and distal links is restricted to the range of 30 to 150°, thereby only including dexterity values in this adjusted range. Maximum global dexterity is 0.722 and obtained with a ratio of 0.743. Despite this, a ratio of 1 is preferable because it greatly simplifies kinematic computing. Indeed, this ratio results in significantly simpler forward kinematic and Jacobian matrix. Moreover, in the 2D workspace, radial values further from the origin hold greater significance as they cover more space upon rotation, thereby reinforcing the preference for a ratio of 1 over 0.743, as depicted by the more distant peak of this ratio in Figure 3. Additionally, the difference with the maximum global dexterity is only 16%, which is an acceptable compromise.

### Workspace

The user workspace is defined as the reachable workspace of a user’s arm in Cartesian coordinates. This workspace must fit completely inside the robot’s reachable workspace. Furthermore, the dimensions of the robot must be adjusted to ensure good dexterity within the user workspace.

**Figure 5.**
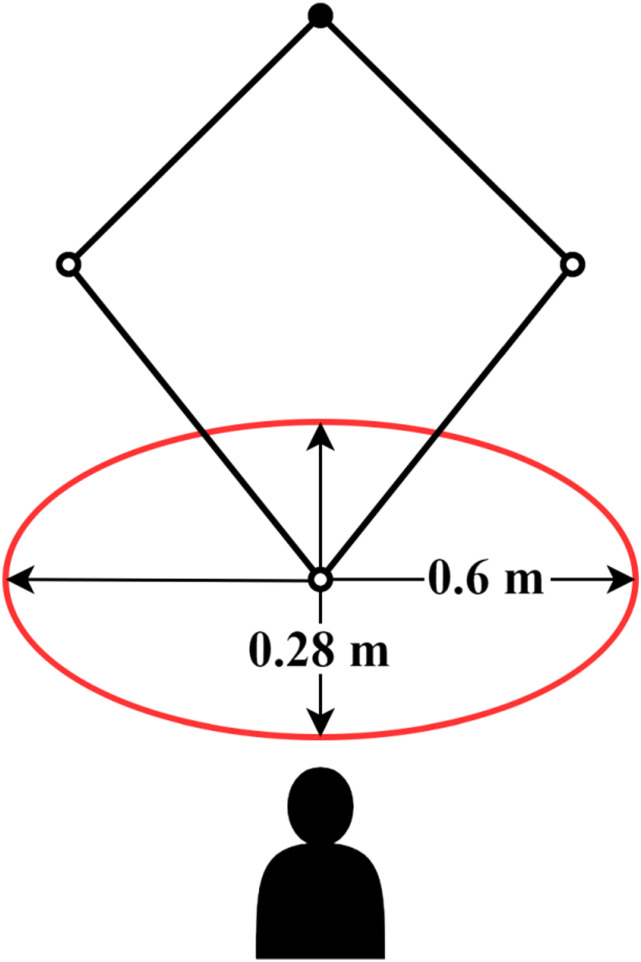
Representation of the user workspace with the placement of the robot and user.

A workspace analysis of the upper limb for a planar robot determined the range of motion for seated unforced movement using arm geometry.^19^ The results indicated a range of 254 mm along the sagittal axis and 645 mm along the frontal axis. Therefore, for this study, the user workspace is defined as an ellipse of minor axis of 280 mm and major axis of 600 mm as presented in Figure 5.

**Figure 6.**
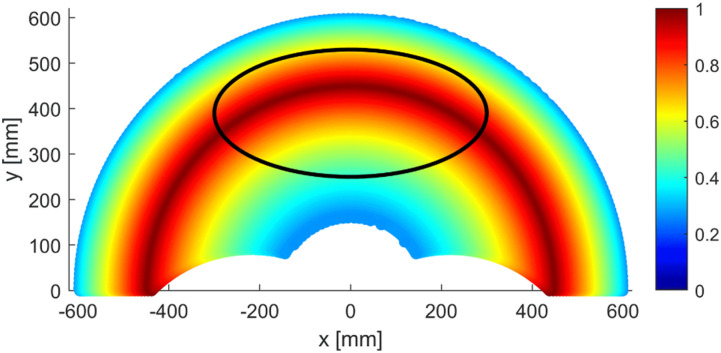
Dexterity value in the robot’s reachable workspace for the chosen lengths and constrains. The user workspace ellipse is drawn over.

Using the ratio *L*_1_/*L*_2_ of 1, a radial reach of 600 mm is chosen, which translate to link lengths of 300 mm. Mechanical stops are introduced in the design to mechanically prevent the robot from getting close to singularities as previously stated. Similarly, angles *θ*_1_ and *θ*_2_ are confined within the range of −45 to 225°, as only the +y portion of the workspace is required. The resulting workspace is presented in Figure 6. The user ellipse is placed to maximize global dexterity, which results in a value over 0.8 for most of the centre and an average of 0.76 across the ellipse.

## Design

### Control method and actuation

Impedance and admittance control are very popular options for physical Human-Robot interaction. Both control schemes are defined by force control through simulating spring, damping and inertia using the equation of a virtual object. Impedance control regulates the end effector’s apparent mechanical impedance and is defined by dynamic control relating force and position. Consequently, it is well suited for applications with high motor transparency, typically in scenarios with low inertia or the need for high compliance. On the other hand, admittance control modulates the end effector’s compliance to external forces, necessitating force sensing. It is preferred for high load or inertia applications, or situations with significant friction.

Given the objective of accompanying the user’s movement, impedance control is favoured over admittance control in the current situation. Indeed, a backdriveable robot coupled with impedance control can allow the user to move freely. Conversely, with admittance control, the user’s movement is realized by the robot following force input by the user, which requires expensive sensors and can introduce delay in movement. This small delay can be detrimental to the rehabilitation process. Additionally, given the nature of the robot, impedance control complements the choice of a parallel robot architecture as well as the need for transportability since the common goal is low end-effector inertia and mass.

The challenge with impedance control is the need for low reduction and high torque density motor. The robot must be backdriveable and have high bandwidth. Conversely, high mechanical impedance introduces friction, which can affect the reproduction of forces, and can cause instability in high-speed impedance control.^20^ However, direct drive motors are characterized by low torque density along with being expensive, bulky and heavy. Indeed, most electromagnetic actuators peak in power at high speed and low torque, which does not meet the requirements for position and force control.

The MIT-cheetah robot addressed this very issue^20^ with proprioceptive motors, which were devised by integrating high-radius BLDC (brushless DC) motors with a 6:1 single-stage planetary gear reduction. This approach aimed to optimize torque density while minimizing the impact on motor transparency. Subsequent iterations of the project aimed at cost reduction by employing high-performance motors commonly used in RC drones and airplanes, which are produced in large quantities.^21^

**Figure 7.**
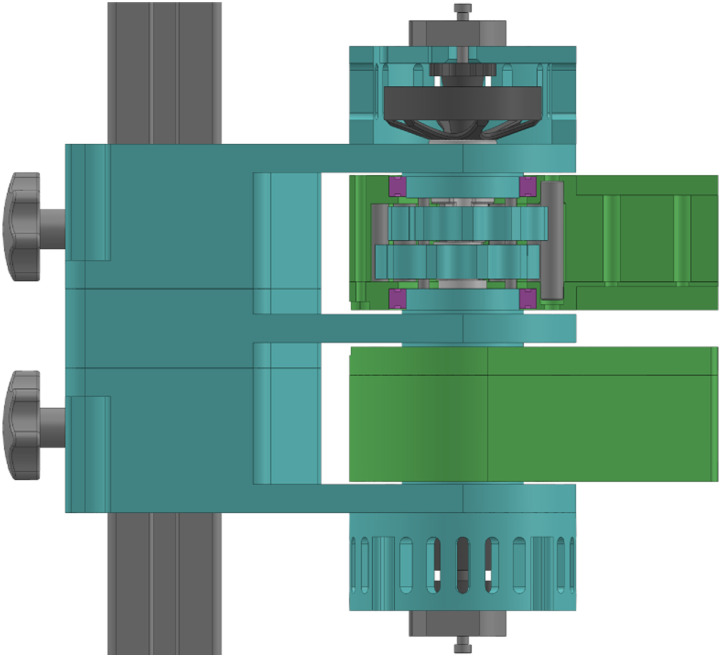
Partial cut view of the motor assembly. The motor drives the eccentric shaft of the cycloidal reduction. The outer casing of the gearbox acts as the output.

A similar approach is used for this robot as shown in Figure 7, which utilizes off-the-shelf RC drone motor and single stage reduction. The chosen motor is a 48V 14 pole pairs BLDC outrunner motor (T-Motor MN5008 Antigravity). While various reduction options were considered, including spur gear and planetary gear reductions, a cycloidal gearbox was selected due to its superior reduction density compared to gear-based alternatives, albeit at the expense of increased complexity. Similar to planetary gear reduction, the cycloidal gearbox distributes the load more evenly, thereby enhancing shock resistance. The result is an 11:1 gearbox with equivalent radius to the motor.

### Assembly description

**Figure 8.**
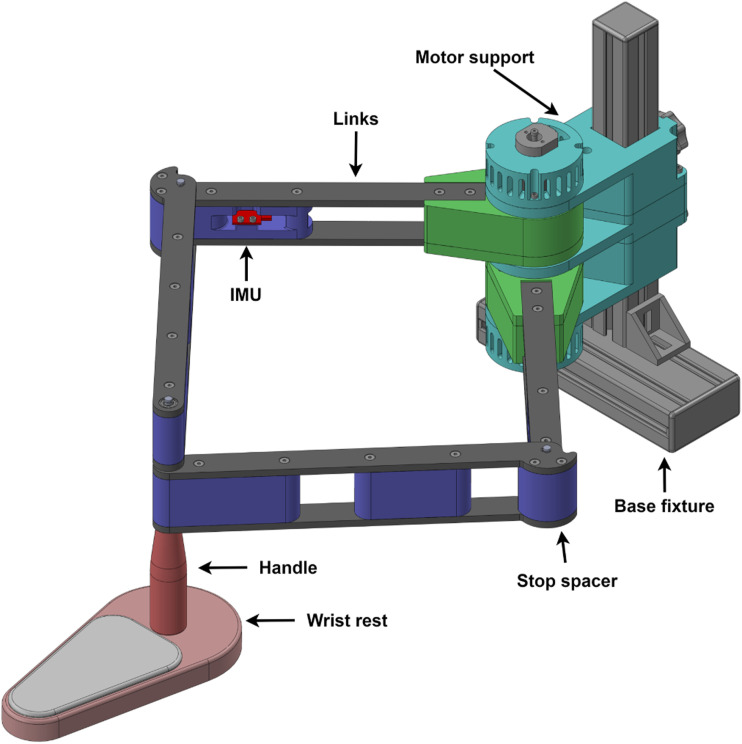
CAD model of the prototype.

Following the chosen architecture and optimization process, the prototype shown in Figure 8 was developed. The assembly is divided in four sections: base fixture, motor support, links and end effector. The base is comprised of standard aluminum T-framing extrusions, with a double extrusion at the bottom which can be clamped to the end of a table, securing the robot in place. The motor support is affixed to the base at the desired height, featuring handles at the rear for ease of adjustment.

The links are constructed using a combination of 3D printed spacers and easily machinable ABS plates. The spacers located at the rotational link between *L*_1_ and *L*_2_ also act as stops to prevent singular configurations. The end effector is composed of a handle and wrist rest. The wrist rest is designed as a cushioned plate equipped with rollers, which efficiently transfer the vertical load to the table rather than the robot. With the exception of a few metal rods and purchased components, the entire assembly is either 3D printed or fabricated from machined ABS plates on a 3-axis CNC machine.

The motor support contains two motors each coupled to a proximal link via an output fixture. This output fixture allows the load to be transferred to the motor support rather than to the motor rotor while also housing the cycloidal gearbox. This design enables a compact motor assembly and allows both motors to be coaxial along their rotation axis. Thus, the whole motor assembly acts as a fixed pivot instead of a fixed link as outlined in the kinematic section. Shoulder screws installed at the rear of the motor enable the incorporation of incremental encoders (ATM103-V). Additionally, IMUs (inertial measurement unit) (Sparkfun LSM9DS1) are installed at the end of each proximal link to measure tangential acceleration.

**Figure 9.**
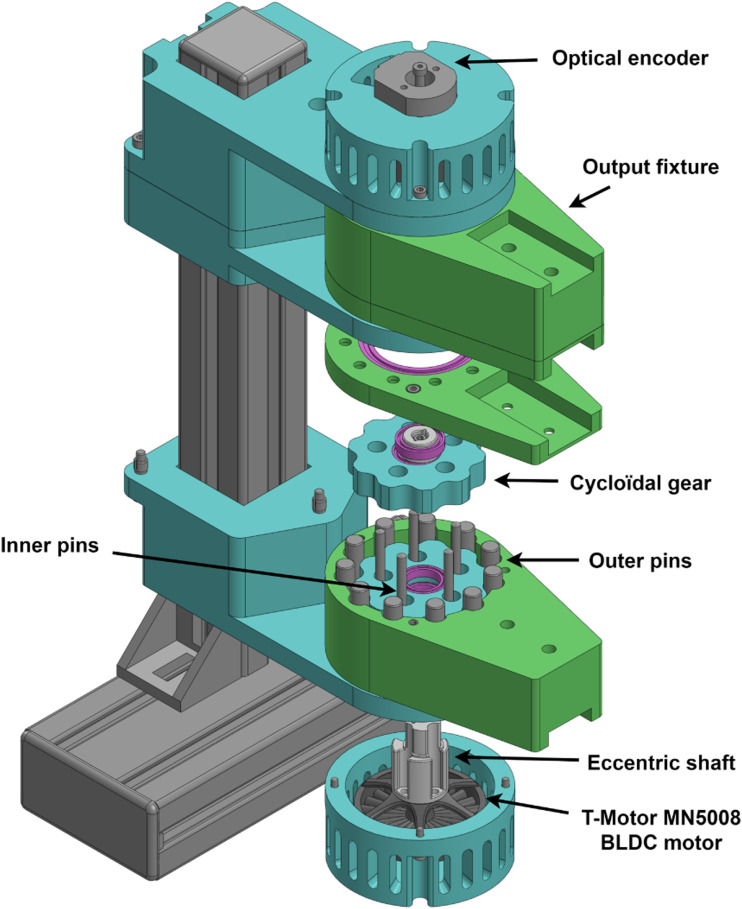
Exploded view of the motor assembly.

The cycloidal gearbox, presented in Figure 9, is composed of outer pins, inner pins, cycloidal gears and an eccentric shaft. The motion of the eccentric shaft, driven by the motor, allows the centre of both gears to rotate. With each revolution of the gears, the output shifts by one outer pin. Indeed, unlike traditional cycloidal gearboxes, the inner pins are fixed and the outer pins are rotating, which allows the assembly to be compact with a reduction ratio of 11:1. Given the eccentric nature of the gearbox, two gears offset by 180° are used in order to mitigate rotating imbalance as well as reduce backlash.

## Control

### Motor control

Many such algorithms exist, varying in complexity, with trapezoidal being the simplest to program. However, this simplicity often results in drawbacks such as noisy operation, torque ripple, and reduced torque output. Consequently, trapezoidal commutation is primarily utilized in sensorless speed control applications, such as RC drones.

To achieve maximum torque and smooth motion, FOC (Field Oriented Control) is used. This algorithm differentiates itself with a current loop and a rotation reference frame to independently regulate motor flux and torque. This, however, makes the FOC algorithm complex and mathematically intensive. As a result, a dedicated microcontroller is typically assigned to drive each motor implementing FOC. Fortunately, numerous FOC libraries are available in the open-source community, such as SimpleFOC^22^ for the Arduino platform. In this case, the STM32 environment is used because of its high performance and user-friendly setup package.

### Control schematic

The control system is structured into three main components: the master board, the slave boards and the user interface. The slave boards are composed of a shield driver (X-NUCLEO-IHM07M1) and a control board (NUCLEO-F302R8) tasked with executing the FOC algorithm in torque control mode. These units communicate with the master board via the RS-485 communication protocol. The master board assumes responsibility for overall robot control, consolidating data, computing kinematics, and issuing impedance-based torque commands to the motors at a frequency of 250 Hz. Both the boards and the drives are powered by a 12 V DC power supply with a capacity of 120 W. The master board can be connected to a computer via USB, enabling control of the robot through a user interface on the computer screen. This interface serves as a visual platform for conducting exercises. The communication between the computer and the board utilizes the USART protocol.

**Figure 10.**
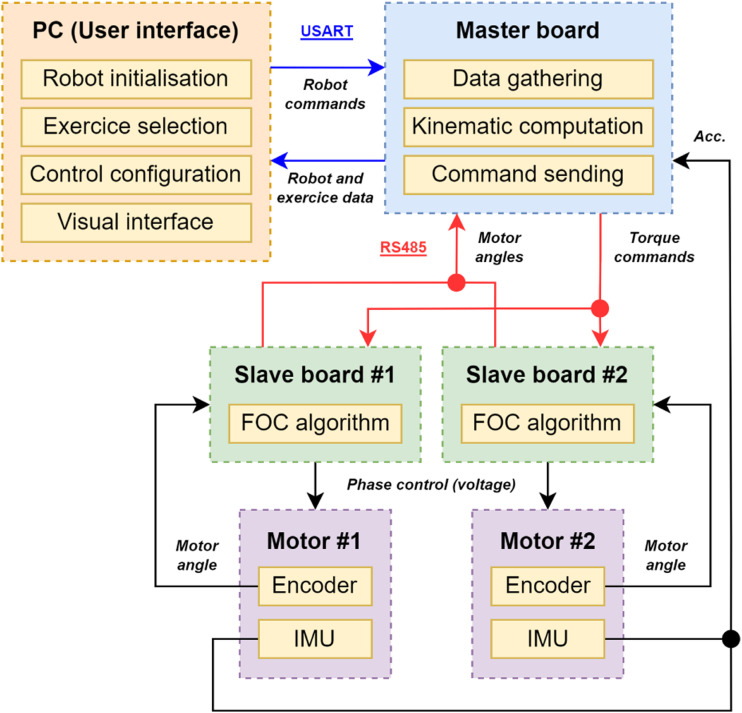
Control schematic of the robot. Communication protocols are identified through colored arrows.

**Figure 11.**
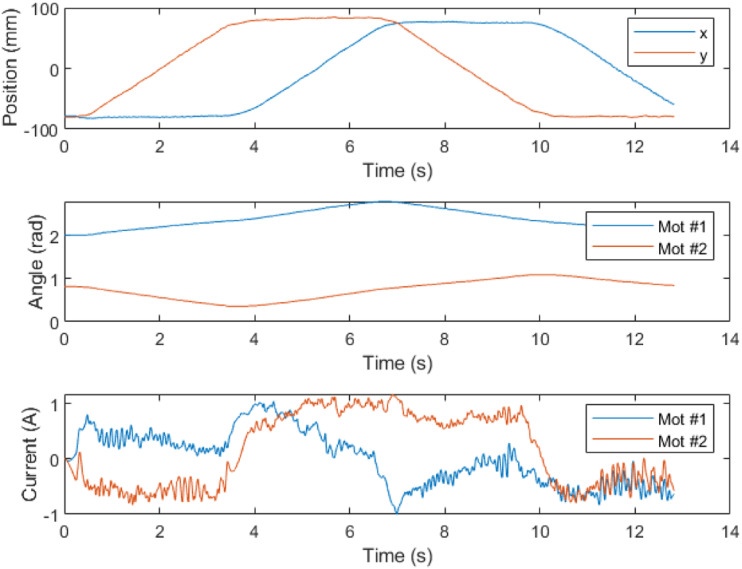
Position, angle and current data for a square trajectory realised by the robot.

Both motors are equipped with added incremental encoders, connected to their respective driver cells, providing angle feedback. As previously stated, the IMUs supply acceleration feedback to the master board. A comprehensive schematic of the control system is presented in Figure 10. Figure 11 presents testing data for a simple square trajectory realized by the robot.

### User interface

**Figure 12.**
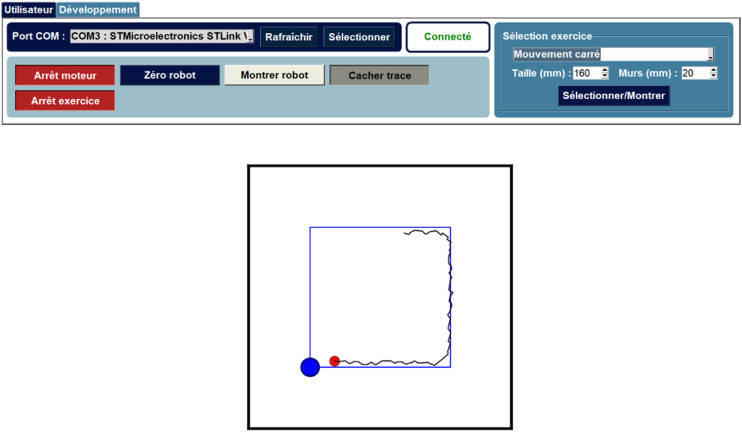
User interface for the robot in its functional state during a square trajectory.

The user interface, depicted in Figure 12, provides users with several functionalities. It enables the initialization of the robot by activating the motors and zeroing its position. An additional stop piece must be used in order to obtain the right configuration. On the right side of the interface, users can select exercises and their parameters. Various options, such as displaying the user’s track and the robot links, can be toggled as well. Furthermore, a dedicated tab allows specialists to fine-tune the impedance values for different exercise types.

### Control mode

**Figure 13.**
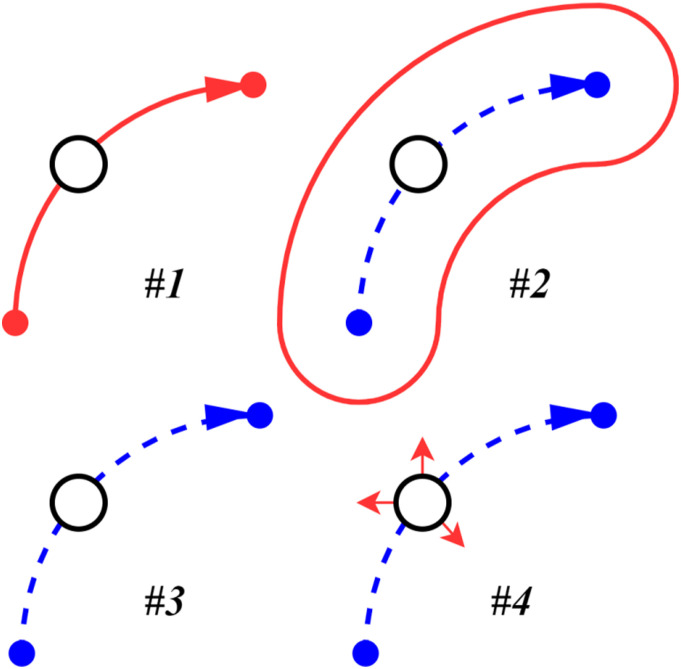
Representation of the different levels of assistance ranging from completely assisted (#1), virtual wall assistance (#2), free movement (#3) and free movement with perturbation (#4).

As outlined in the introduction of this paper, the aim of this robot is to offer upper-limb exercises to simulate ADL movements with different levels of assistance. These levels are illustrated in Figure 13 and are as follows:

1. Movement realized by the robot.
2. Movement guided by virtual walls.
3. Free movement.
4. Free movement with random perturbations.

**Figure 14.**
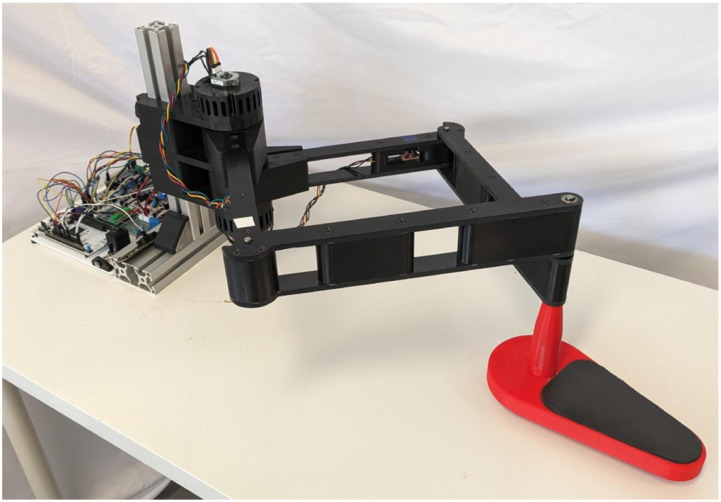
Prototype of the robot.

For the first 3 levels, an option to reduce the user’s perturbation can be toggled. The goal is to implement exercises such as point-to-point movement, drawing shapes and interactive games. While some exercises have been implemented in the robot with the first two levels of assistance, the algorithms are not presented in this paper. The working prototype is shown in Figure 14.

## Conclusion

This paper focused on the design of an affordable and easy to install planar robot with the aim to provide rehabilitation exercises at home. A prototype was developed through kinematic analysis and optimization of link lengths. Motor assemblies utilizing RC drone motors, along with compact cycloidal gearboxes, were designed for impedance control. Additionally, robot control was implemented via a user interface.

Future work will consist of programming various levels of assistance, such as guided movement, virtual walls, and spasm damping, into the robot’s control system. Subsequent steps include programming exercises and games for user testing. Based on feedback obtained from future clinical validation, further iterations of the robot will be developed.
